# Borax in Epilepsy

**Published:** 1892-06

**Authors:** 


BORAX IN EPILEPSY.—Dr. Maiset has obtained excellent results in the treatment of epilepsy by borate of soda. He finds it more efficacious than the bromides in symptomatic epilepsy, but less so in the nervous forms.—*Le Progres Medical.*

